# Suppression of Hepatocyte Nuclear Factor 4 α by Long-term Infection of Hepatitis B Virus Contributes to Tumor Cell Proliferation

**DOI:** 10.3390/ijms21030948

**Published:** 2020-01-31

**Authors:** Soree Park, Yea Na Ha, Mehrangiz Dezhbord, Ah Ram Lee, Eun-Sook Park, Yong Kwang Park, Juhee Won, Na Yeon Kim, Soo Yeun Choo, Jae Jin Shin, Chang Hyun Ahn, Kyun-Hwan Kim

**Affiliations:** Department of Pharmacology, Center for Cancer Research and Diagnostic Medicine, IBST, School of Medicine, Konkuk University, Seoul 05029, Korea; rhd37@naver.com (S.P.); clean1701@naver.com (Y.N.H.); asal_dejbord@yahoo.com (M.D.); ahram2g@naver.com (A.R.L.); espark97@gmail.com (E.-S.P.); yk1029@korea.kr (Y.K.P.); 1wonjuhee@hanmail.net (J.W.); michaela3310@naver.com (N.Y.K.); michellechoo@naver.com (S.Y.C.); 362whe12@naver.com (J.J.S.); quek689@gmail.com (C.H.A.)

**Keywords:** Hepatitis B virus, hepatocyte nuclear factor 4 α, long-term infection, ERK signaling pathway

## Abstract

Hepatitis B virus (HBV) infection is a major factor in the development of various liver diseases such as hepatocellular carcinoma (HCC). Among HBV encoded proteins, HBV X protein (HBx) is known to play a key role in the development of HCC. Hepatocyte nuclear factor 4α (HNF4α) is a nuclear transcription factor which is critical for hepatocyte differentiation. However, the expression level as well as its regulatory mechanism in HBV infection have yet to be clarified. Here, we observed the suppression of HNF4α in cells which stably express HBV whole genome or HBx protein alone, while transient transfection of HBV replicon or HBx plasmid had no effect on the HNF4α level. Importantly, in the stable HBV- or HBx-expressing hepatocytes, the downregulated level of HNF4α was restored by inhibiting the ERK signaling pathway. Our data show that HNF4α was suppressed during long-term HBV infection in cultured HepG2-NTCP cells as well as in a mouse model following hydrodynamic injection of pAAV-HBV or in mice intravenously infected with rAAV-HBV. Importantly, HNF4α downregulation increased cell proliferation, which contributed to the formation and development of tumor in xenograft nude mice. The data presented here provide proof of the effect of HBV infection in manipulating the HNF4α regulatory pathway in HCC development.

## 1. Introduction

Chronic Hepatitis B Virus (HBV) infection is one of the global health problems. The World Health Organization estimates that approximately 240 million people worldwide are chronically infected, despite the availability of an effective HBV vaccination program. Chronic Hepatitis B (CHB) infection is considered as a serious risk factor in the development of liver cirrhosis and hepatocellular carcinoma (HCC) [1]. 

As the fifth most common type of human liver cancer, malignant HCC is the cause of many cancer-related deaths globally [2]. Over 500,000 people worldwide are affected by HCC and the incidence is increasing [3]. The lack of early diagnostic markers and effective therapies are two significant impediments to controlling the disease. HCC is mostly prevalent in Africa and Eastern Asia due to chronic HBV infection [2]. However, the mechanism by which HBV infection contributes to the development of HCC remains unclear. 

HBV is an enveloped virus and a member of the Hepadnaviridae family, that contains a partially double-stranded DNA genome 3.2 kb in length. The HBV genome contains four genes (core, surface, X, and polymerase). HBV X protein (HBx) is a small trans-activator protein with a molecular mass of 16.5 kDa which activates the transcription of HBV [4,5,6]. Increasing evidence suggests that the HBx protein plays an important role in the development of HCC [7,8].

Hepatocyte nuclear factor 4 α (HNF4α) is a protein which belongs to the nuclear receptor superfamily and binds to DNA as a homodimer. HNF4α is expressed in hepatocytes and plays a critical role in the transcription and expression of many liver-specific genes [9,10]. It is also essential for the hepatic epithelium formation during embryonic development. Moreover, HNF4α is known as a key tumor suppressor that inhibits the progression of HCC. Thus, there are several studies that HNF4α expression level is suppressed in human HCC tissue compared to other adjacent noncancerous tissues [11,12,13].

Previous studies have examined the expression level of HNF4α during HBV infection. However, HNF4α expression level varies among different studies. HNF4α was shown to be downregulated in rat primary hepatocyte transfected with HBV plasmid [14], and in a human hepatoma cell line transfected with HBV plasmid [15]. In these cases, the level of HNF4α protein was examined in the early stage of HBV infection. Contrarily to these reports, the expression of HNF4α was enhanced in chronic HBV-infected patients [16]. Thus, in HBV infection, the level of HNF4α has yet to be clarified. 

Previous reports have shown that the rat sarcoma virus (Ras)/Raf/MEK/ERK signaling pathway, the c-Jun N-terminal kinase (JNK) pathway and the phosphoinositide 3-kinase (PI3K)/AKT signaling pathway are the major signaling pathways regulating cell proliferation and differentiation in HCC cells [17,18]. Among these, the ERK signaling pathway is one of the main pathways in HCC which is associated with cell proliferation [19] and previous studies have revealed that HBx activates the ERK signaling pathway [20,21,22]. 

Previously, HNF4α suppression was shown to be associated with the AKT signaling pathway [14] in primary rat hepatocyte. However, the mechanism of HNF4α suppression in human hepatocytes has not yet been clearly identified. The aim of this study was to investigate the effect of HBV on the expression of HNF4α in human hepatocytes and the role of HNF4α suppression in tumor cell proliferation in vivo and in vitro. We found that the expression level of HNF4α was suppressed by HBV only in long-term infection. Our data demonstrate that HNF4α downregulation contributes to the formation and development of tumor in xenograft nude mice. 

## 2. Results

### 2.1. Expression of HNF4α Protein is Significantly Downregulated in Long-term Expression of HBV

**Figure 1:**
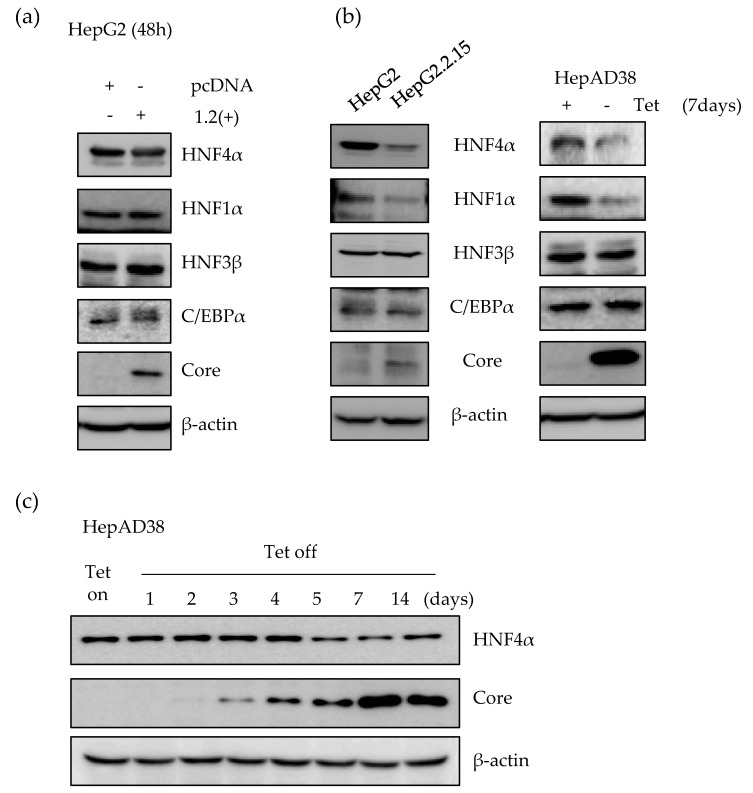
Expression level of HNF4α in early stage and long-term of HBV expression in vitro. (**a**) HBV 1.2mer (1.2(+)) plasmid was transfected into the HepG2 cells. Cells were then harvested at 48 h after transfection. The level of proteins was analyzed by Western blot. (**b**) Expression levels of transcription factors in HepG2, HepG2.2.15, and HepAD38 cell lines was determined by Western blot. In HepAD38 cell line, the complete medium without tetracycline, was used for 7 days in order to induce HBV production. (**c**) The expression level of HNF4α in HepAD38 cell line at indicated time points was determined in the absence of tetracycline. The data represent the results from three independent experiments.

First, we determined the expression level of HNF4α using transient as well as stable HBV expression human hepatoma cell lines. We measured the level of HNF4α after transfection with HBV1.2mer plasmid in HepG2 cell line. At the transiently transfected state, there was no significant effect on the level of HNF4α up to 2 days post transfection (Figure 1a). However, in the HepG2.2.15 cell line which stably expresses the HBV genome from chromosomally integrated HBV sequence, the level of HNF4α was suppressed as compared to the parental HepG2 cell (Figure 1b). In addition, a slight reduction was observed in the level of HNF1α after stable expression of HBV. In contrast, the expression level of other nuclear transcription factors such as HNF3β, and C/EBPα did not alter after the transient or stable expression of the HBV genome (Figure 1a,b).

In the HepAD38 cell line, HBV genome expression is under the control of a tetracycline inducible promoter. Therefore, tetracycline withdrawal induces viral RNA and secretion of virus-like particles into the supernatants. After 7 days of tetracycline removal, the level of HNF4α was suppressed in the induced HepAD38 cell line compared to the control cells, maintained in tetracycline containing medium (Figure 1b). 

As shown in panel C, the level of HBV core protein as a HBV expression marker was measured up to 2 weeks post induction. The core protein formation started from 48 to 72 h after tetracycline removal and was gradually accumulated in the cells. The amount of HNF4α protein, however, remained constant up to 4 days post HBV induction and between days 5 and 14, there was a considerable reduction in the signal intensity of the HNF4α protein. An inverse correlation between the expression level of HBV and HNF4α was observed in the HepAD38 cell line (Figure 1c). Therefore, at later time points following the continuous expression of HBV, the level of HNF4α began to decrease, indicating that the level of HNF4α decreased in long-term expression of HBV.

### 2.2. HBx Protein is Responsible for the Suppression of HNF4α in Long-term HBV Expression

**Figure 2:**
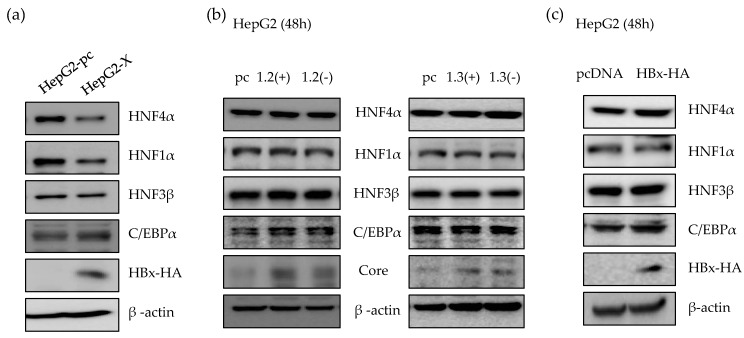
Suppression of HNF4α via HBx protein in long-term expression of HBV. (**a**) The level of proteins in HepG2-pc, HepG2-X cell lines were analyzed by Western blot. (**b**,**c**) HepG2 cells were transfected with 2 µg of indicated plasmids per well in a 6-well plate and harvested after 48 h. The levels of proteins were determined by Western blotting. The data represent the results from three independent experiments.

It is known that HBx is a regulatory protein and associated with the expression of HNF4α in rat primary hepatocyte which does not support HBV infection [14]. Therefore, we investigated the effect of HBx protein on HNF4α level in human hepatocytes. As a long-term HBx expression model, we generated a HepG2-X stable cell line which stably overexpress HBx protein. As shown in Figure 2a, the level of HNF4α protein in HepG2-X stable cell line was suppressed compared to the control, HepG2-pc cell. We next determined the HNF4α level in the presence or absence of HBx protein in a transient transfection using replication-competent wild type HBV 1.2mer (1.2(+)) and HBV 1.2mer X-null (1.2(-)) plasmids. HBV 1.3mer (1.3(+)) plasmid contains double copy of the HBx gene and were used in parallel along with the HBV 1.3mer X-null plasmid (1.3(-)). In a transient state, there were no changes in the level of HNF4α after 48 h of transfection with each plasmid (Figure 2b). In addition, overexpressing HBx by transfection of HBx plasmid to HepG2 cells had no effect on the level of HNF4α (Figure 2c). As a result, suppression of HNF4α was only observed in long-term of expression of the HBx protein. 

### 2.3. ERK Signaling Pathway is Associated with the Downregulation of HNF4α

**Figure 3:**
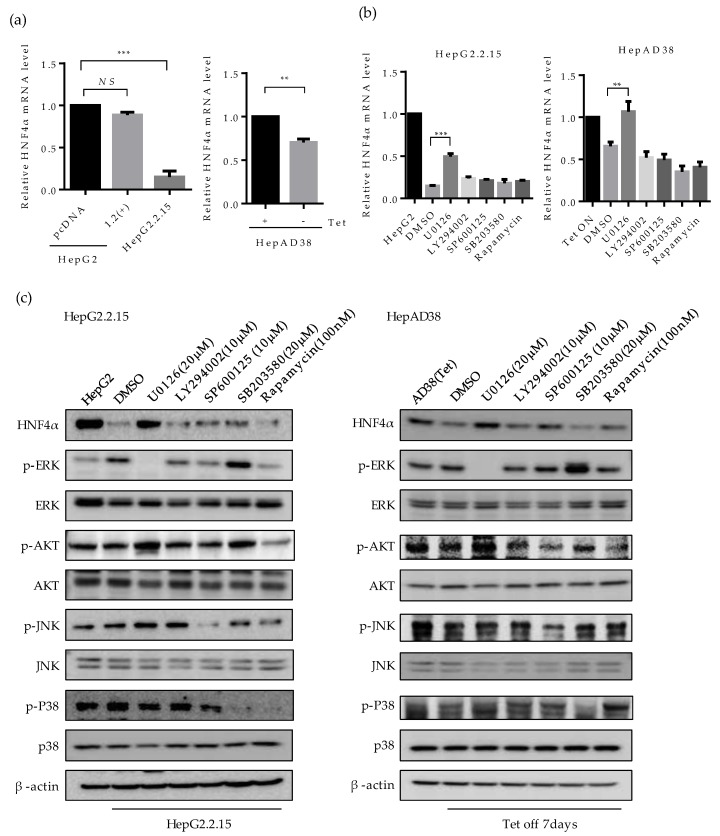

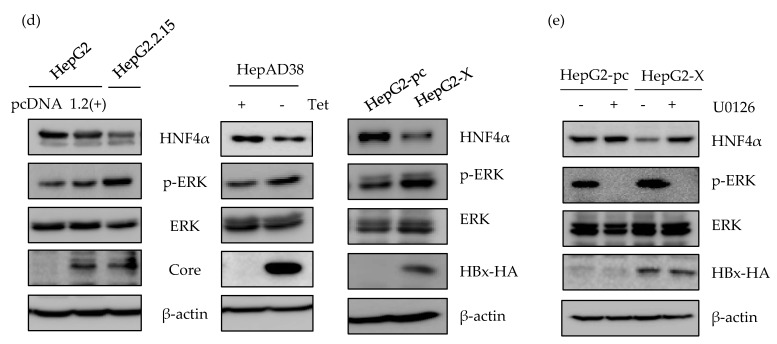
Involvement of ERK signaling pathway in suppression of HNF4α. (**a**) The expression level of HNF4α was analyzed by qRT-PCR analysis in human liver cancer cell lines. (**b**) HepG2.2.15 and HepAD38 cells were treated with U0126, LY294002, and SP600125 (10 μM), 20 μM of SB203580 and 100 nM of Rapamycin at 24 h before harvest. The relative level of HNF4α mRNA was determined by qRT-PCR. ** *p* < 0.01, *** *p* < 0.001. (**c**,**d**) The activation of various signaling pathways and HNF4α expression were analyzed by Western blot in HepG2, HepG2.2.15, HepAD38, HepG2-pc, and HepG2-X. Inhibitors were treated as described in (**b**). (**e**) The expression levels of HNF4α, p-ERK, ERK, and HBx in HepG2-X and HepG2-pc cells were measured by Western blot following treatment with or without ERK inhibitor, U0126 (10 μM). The data represent the results from three independent experiments.

To examine the mechanism of HNF4α suppression, we investigated the mRNA level of HNF4α by quantitative real-time PCR (Figure 3a). The mRNA level of HNF4α decreased in HepG2.2.15 compared to HepG2, but not in HBV 1.2mer transfected cells (Figure 3a, left). Similarly, at one-week post HBV induction the mRNA level of HNF4α reduced in HepAD38 cells as compared to the cells maintained with tetracycline (Figure 3a, right). These results are consistent with the results of protein level (Figure 1a,b), suggesting that the suppression of HNF4α occurs at the transcriptional level.

Having shown that HNF4α is suppressed at the transcriptional level, we then investigated the signaling pathway that is associated with this suppression by interrupting different signaling pathways. Accordingly, the inhibitors for ERK (U0126), AKT (LY294002, Rapamycin), JNK (SP600125), p38 (SB203580), and mTOR/AKT (Rapamycin) were treated in HepG2.2.15 and HepAD38 cells. The suppressed mRNA level of HNF4α was recovered only following the inhibition of ERK signaling pathway (U0126) in both cell lines (Figure 3b, left and right). Other signaling pathway inhibitors had no significant effect on HNF4α expression level. 

The level of HNF4α protein were measured in parallel. Suppression of HNF4α was only restored by inhibiting the ERK signaling pathway in HepG2.2.15 (Figure 3c, left panel), and HepAD38 (Figure 3c, right panel). Successful suppression of each signaling pathway by the selected signal inhibitor was confirmed through measurement of the phosphorylated form of each target protein (p-ERK, p-AKT, p-JNK, and p-P38). Moreover, the unphosphorylated form of target proteins were determined as a proof of activation of each signaling pathway in the two HBV stable cell lines (Figure 3c, right and left panels). 

The activation of ERK was compared with the transiently expressed HBV and further confirmed in HepG2, HepG2-pc, and HepG2-X cells (Figure 3d). The p-ERK-dependent suppression of HNF4α was only observed in stable cell lines. Moreover, the suppressed level of HNF4α was recovered by inhibiting the ERK signaling pathway (U0126) in HepG2-X stable cells (Figure 3e). The inhibition of ERK was confirmed by measuring phosphorylated ERK. Therefore, these results suggest that HBx downregulates HNF4α at the transcriptional level through the ERK signaling pathway.

### 2.4. HNF4α Expression Is Suppressed in Long-term Expression of HBV in Mice

**Figure 4:**
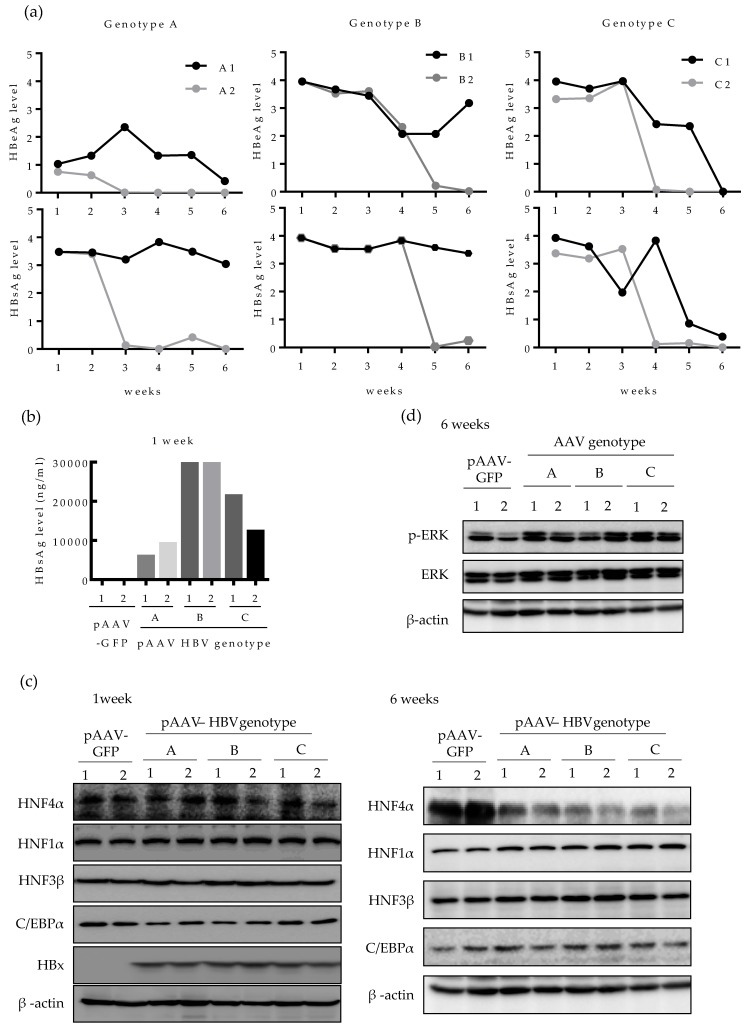
Long-term HBV infection suppressed HNF4α expression in mice liver tissues. Plasmids for pAAV-GFP (*n* = 2) or pAAV-HBV (*n* = 6) (genotype A, B, or C) (12.5 μg each) were hydrodynamically injected in 6 weeks old C56BL/6 mice. (**a**) The relative level of HBsAg and HBeAg in mouse serum were measured weekly by ELISA. (**b**) At 1 week after hydrodynamic injection, the level of HBsAg in serum was quantified. (**c**) At 1 and 6 weeks after hydrodynamic injection with pAAV-GFP or pAAV-HBV (genotype A, B, or C), the level of indicated proteins in mouse livers were determined by Western blotting. (**d**) The activation of ERK was determined by Western blotting of phosphorylated ERK after 6 weeks of HBV expression.

We then investigated whether the level of HNF4α is also downregulated by HBV in vivo. Expression of HBV in mouse liver was done by in vivo transfection, as previously described [23]. The 6 weeks aged C57BL/6 mice were hydrodynamically injected with a number of plasmids harboring different HBV genotypes (A, B, and C) and the levels of HBeAg and HBsAg in mice serum were regularly measured up to six weeks post infection (Figure 4a). The relative level of HBeAg and HBsAg varied between the two mice infected with same genotype (A1, A2; B1, B2 and C1, C2) and among the mice infected with different HBV genotypes. Compared to mice injected with genotype A HBV, the levels of HBeAg and HBsAg lasted longer in mice infected with genotypes B and C. Particularly, in genotype A-infected mice, HBeAg level was lower than that of other genotypes and fell sharply up to the end point of infection course (six weeks) (Figure 4a). To compare the quantitative level of HBsAg between genotypes, the level of HBsAg in mice serum was quantified at one week post infection. In line with the data in Figure 4a, mice injected with pAAV HBV genotype B (B1 and B2), exhibited the highest HBsAg level at one-week post infection (30 μg/mL) whereas genotype A-infected mice (A1 and A2) showed the lowest HBsAg level (10 μg/mL) (Figure 4b). 

Next, we analyzed the HNF4α protein level in the mouse liver tissues. After one week of HBV infection, the level of HNF4α remained constant among all the three HBV genotypes tested (Figure 4c). After 6 weeks of infection, however, the level of HNF4α was strongly suppressed in the livers of all infected mice (Figure 4c, right). The levels of other nuclear factors were not changed during one to six weeks. At 6 weeks post infection, the level of HBx protein was undetectable in all HBV-infected mice by Western blot, implying that the effect of HBx in reduction of HNF4α during long-term HBV infection is cumulative and long-lasting. (Figure 4c).

Considering that the suppression of HNF4α was associated with the ERK signaling pathway in human hepatoma cell lines, we then investigated the level of activated ERK in mice tissues. Western blotting showed that after 6 weeks of HBV infection, p-ERK protein level was upregulated in most liver tissues compared to the controls, implying that the ERK pathway was activated in mouse liver tissue (Figure 4d). These results demonstrate that HBV also downregulates HNF4α in long-term infection through ERK-dependent signaling pathway in vivo.

### 2.5. HNF4α Expression Is Suppressed during Long-term HBV Infection in vitro and in vivo

**Figure 5:**
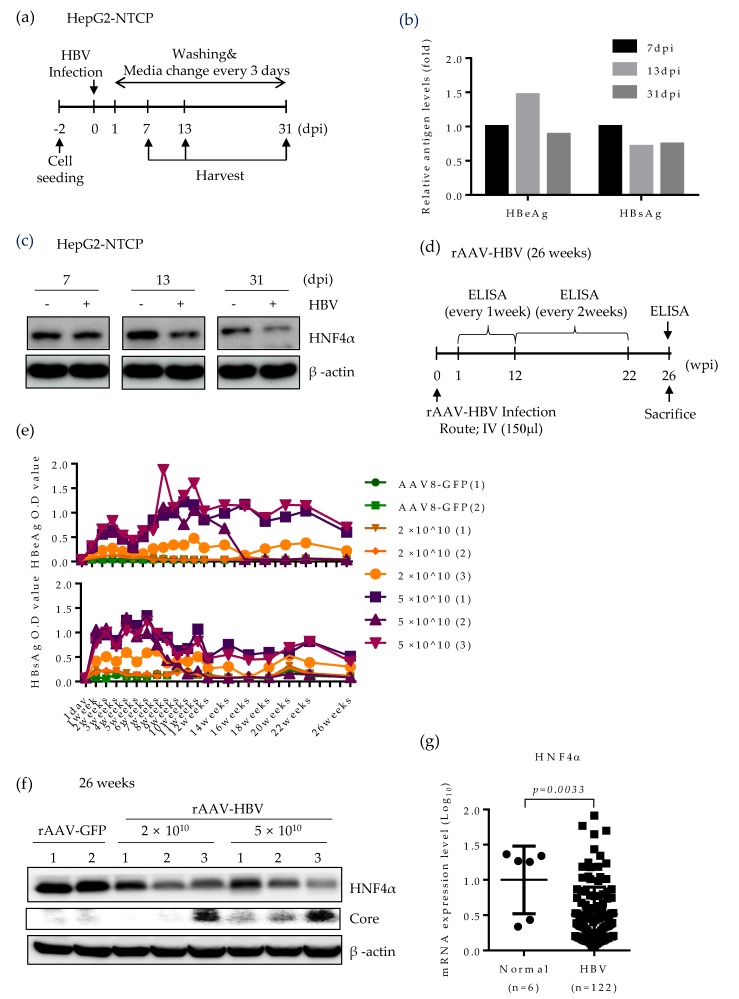
HNF4α expression is suppressed following long-term HBV infection in vitro and in vivo mouse model. (**a**) HepG2-NTCP cells were infected with cell culture derived HBV at 2000 GEq/cell. Cells were maintained in culture medium containing 2.5% DMSO and were harvested at the indicated time points. (**b**) Secreted HBsAg and HBeAg were quantified by immunoassay. (**c**) HNF4α protein level was analyzed by Western blotting. The data represent the results from three independent experiments. (**d**) Recombinant AAV-HBV (rAAV-HBV) (*n* = 6) at different titrations were injected intravenously (IV) (150 μL injection volume) into the tail vein of C57BL/6 mice. rAAV-GFP (*n* = 2) were injected into the tail vein of C57BL/6 mice. (**e**) Serum HBeAg and HBsAg levels from each infected mouse were quantified at indicated time points and plotted. (**f**) Expression levels of HNF4α and HBV core protein in mouse liver tissues at 26 weeks post infection were determined by Western blotting. (**g**) Comparison of expression level of HNF4α mRNA in liver biopsies between normal and CHB patients (GSE83148) obtained from public data. dpi, day post-infection; GEq, genomic equivalent.

Based on the strong suppression of HNF4α in mice expressing HBV, we further seek the effect of HBV infection on HNF4α level using two different infection models. Initially, to determine the role of HBV infection on HNF4α, we performed long-term HBV infection in the HepG2-NTCP cell line which supports the full HBV life cycle [24] according to the experimental scheme in Figure 5a. An early, an intermediate and a late time point (7, 13, and 31 days post infection (dpi), respectively) were selected to analyze the HNF4α level. Successful HBV infection was confirmed by the measurement of HBeAg and HBsAg levels in cell culture supernatant (Figure 5b). Western blot data showed no difference between HNF4α signal intensity in the presence or absence of HBV infection at 7 days post infection (dpi). However, significant reduction of HNF4α protein level was observed at 13 dpi and the HNF4α inhibitory effect of HBV infection was greater at the end of the infection course (31 dpi) (Figure 5c). 

Lastly, HNF4α level was explored in a long-term persistent HBV infection model in mice. Recombinant adeno-associated virus serotype 8 (rAAV)-HBV was intravenously delivered to the mice at two different titrations (Figure 5d). Infected mice were maintained up to 26 weeks post infection. The levels of HBeAg and HBsAg were regularly measured on a weekly basis (Figure 5e). rAAV8-GFP was used as a negative control. Expectedly, blood samples from mice infected with higher HBV titer displayed more HBeAg and HBsAg levels. 

Persistent HBV infection remarkably attenuated the HNF4α protein level in mice livers at 26 weeks post infection (Figure 5f). It seems that the suppressive effect of HBV was not dose-dependent as mice infected with different HBV titers manifested the same reduction level in HNF4α. Nonetheless, HBV core protein was mostly detectable at higher titer of rAAV-HBV. We further analyzed the level of HNF4α in the liver biopsies of CHB patients (GSE83148) [25]. Compared to normal patients, the mRNA level of HNF4α was significantly lower in CHB patients. These results obtained from long-term HBV infection models and public data suggest that persistent HBV infection downregulates HNF4α in vivo.

### 2.6. Suppression of HNF4α Enhances Cell Proliferation in the Sustained Presence of HBV

**Figure 6:**
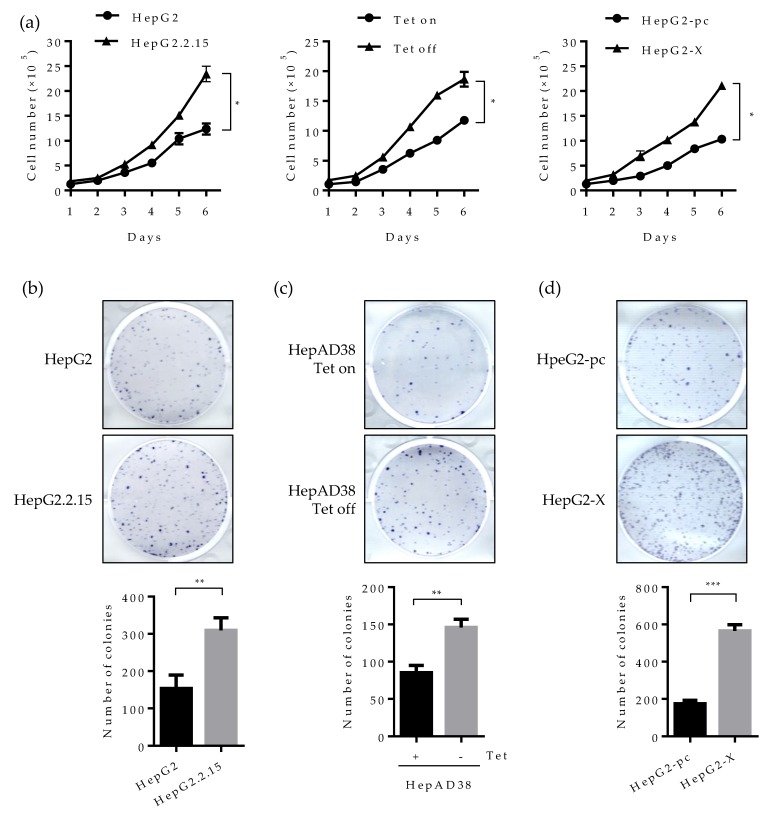

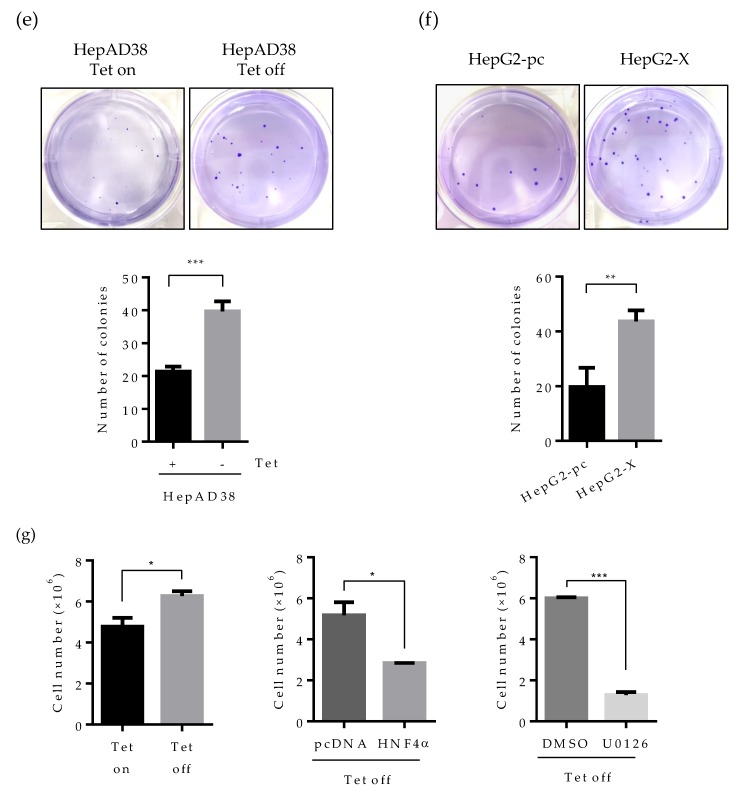
Effect of HBV-induced HNF4α suppression on proliferation of hepatoma cells. (**a**) Cell proliferation assay was performed in human hepatoma cell lines. The indicated cells were seeded at a density of 2 × 10^5^ per well and cultured for 6 days. Cell numbers were counted every day and plotted. (**b**–**d**) The indicated cells were seeded at 2000 cells/well (5000 cells/well for HepG2-pc and HepG2-X cells) and cultured for 4 weeks in complete medium to form colonies. Cell colonies were visualized with crystal violet staining. The number of colonies was counted and plotted. Tet on, with tetracycline; Tet off, without tetracycline. (**e**,**f**) Anchorage-independent cell growth was determined by soft agar assay. HepAD38 (with or without tetracycline) and HepG2 stable (HepG2-pc and HepG2-X) cells were incubated in soft agar plates for 4 weeks. Cells were then visualized by crystal violet staining. The colonies were counted and the data represent the means ± SD of three independent experiments. ** *p* < 0.01. (**g**) Cell proliferation assay in HepAD38 cell lines. A density of the cell was 2 × 10^5^ per 12-well and cultured for 6 days. Tetracycline was removed from indicated samples at the time of seeding. HNF4α plasmid were transfected one day after Tet off. Moreover, the ERK inhibitor, U0126, was treated at a concentration of 20 μM every day. Cells were counted at 4 days post transfection or inhibitor treatment. Cell numbers were analyzed and compared in a bar graph. The data represent the means of results from three independent experiments. * *p* < 0.05; *** *p* < 0.001. The statistical significances were performed by a Student’s *t*-test.

Studies have revealed that HNF4α inhibits cell proliferation in hepatocytes and abolished HNF4a promotes tumor generation in the liver [25]. We, therefore, examined whether the suppressed HNF4α by HBx really promotes cell proliferation in human hepatoma cell lines. As presented in Figure 6a (left), cell number doubled at six days following HBV induction (Tet off) in the HepAD38 cell line. In the HepG2.2.15 cell line, the number of cells increased by two-fold compare to parental HepG2 cell (Figure 6a middle). A similar pattern was observed in the HepG2-X cell line (Figure 6a right). These results suggest that the suppressed HNF4α by HBx enhances cell proliferation.

We then examined cell proliferation by colony forming assay. Cells were seeded at 2 × 10^5^ per well and incubated for 3 weeks. Consistently with the data obtained in Figure 6a, the number of colonies was increased by approximately two-fold in HepG2.2.15 and HepAD38 cell lines (Figure 6b,c). Cell proliferation was more dramatic in the HepG2-X cell line (three-fold increase) (Figure 6d). 

Since the human hepatoma cell lines used in this study are a type of cancer cell lines, cells can grow in an anchorage-independent environment. To assess the effect of HBx-induced HNF4α suppression on anchorage-independent growth, we examined colony formation by soft agar assay. Accordingly, cells were seeded at 1 × 10^4^ per well and incubated for 4 weeks. Then, the colonies were stained for visualization. In the plate containing HBV expressing HepAD38 cells (no tetracycline), colony-forming efficiency increased by two-fold compared to the tetracycline treated control plate (Figure 6e). Likewise, colony-forming efficiency was doubled in HepG2-X cells as compared to vector-transfected control cells (Figure 6f). To further examine the direct effect of HNF4α on the cell proliferation, HBV-expressing HepAD38 cells (Tet off) were transiently transfected with HNF4α and were counted after four days. Compared with cell transfected with pc DNA, the number of cells transfected with HNF4α was significantly decreased from 5.5 × 10^6^ to 2.8 × 10^6^ cells per well (Figure 6g).

In parallel the effect of HBV mediated HNF4α downregulation on the cell proliferation was examine by a pharmacological approach. To do so, HBV-expressing HepAD38 cells were treated with U0126 (ERK inhibitor) at 20 μM in another experimental set and the counted cell numbers were plotted in the bar graph (Figure 6g). The number of cells treated with the ERK inhibitor was decreased by 4.7-fold as compared to the DMSO treated cells. These results suggest that HBx enhances cell proliferation and colony formation in human hepatoma cells through the downregulation of HNF4α.

### 2.7. Effect of HBV-Induced Suppression of HNF4α on Tumor Formation and Growth in Xenograft Mice

**Figure 7:**
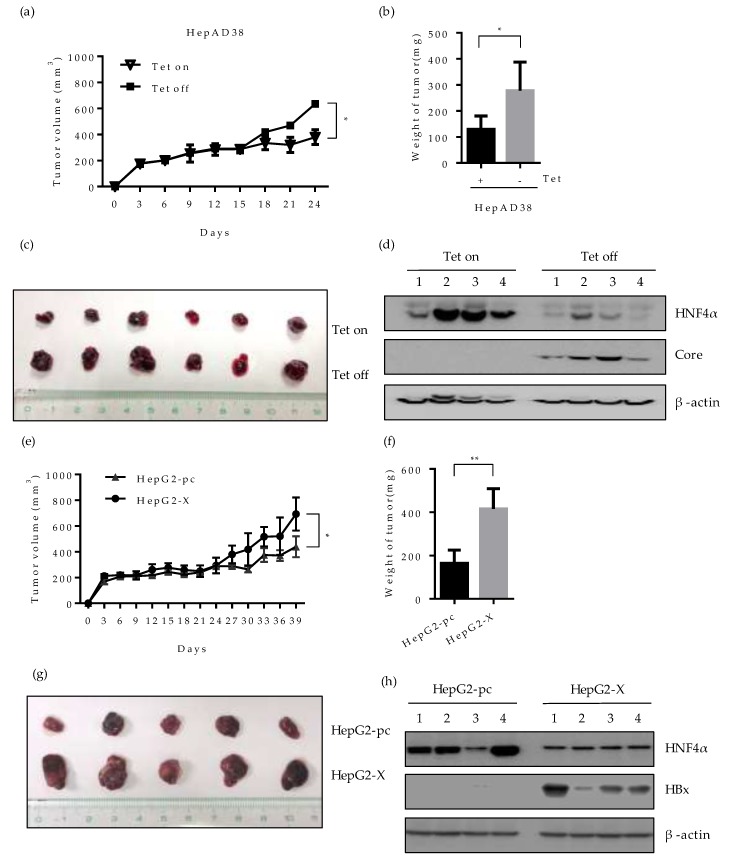
Effect of HBV-induced HNF4α suppression on tumor formation and growth in a xenograft mouse model. HepAD38 (**a**–**d**) or HepG2-X (e-h) cells (1 × 10^7^) were mixed with Matrigel (1:1) and injected subcutaneously (SC) into the nude mice (n=5-6/group). (**a**,**e**) The size of tumors bearing the indicated cells was measured every three days. The volume of the tumor was calculated and is shown in the growth curve. The Tet-on mice were housed with water containing 2 mg/mL tetracycline. Mice were sacrificed after 4 weeks. (**b,f**) Tumor weight after 4 weeks of injection were plotted. * *p* < 0.05. (**c**,**g**) Pictures of xenografted tumors at 4 weeks following SC injection. (**d,h**) The expression levels of HNF4α and HBV core/HBx protein in representative mouse livers were determined by Western blotting at 4 weeks post SC injection.

To evaluate whether the downregulation of HNF4α by HBV would affect the tumorigenicity of hepatoma cells in vivo, we investigated the tumor formation and growth in nude mice bearing HepAD38 or HepG2-X xenograft. HepAD38 cells grown in the presence or absence of tetracycline, were injected subcutaneously (SC) into each BALB/c nude mouse, respectively and the size of tumor was measured every 3 days. The mice injected with HepAD38 (grown with tetracycline) were housed with water containing 2 mg/mL tetracycline to suppress HBV expression and maintain the HNF4α level. Up to 15 days after injection, there were no significant changes in the volume of tumor in mice bearing the HepAD38 xenograft. However, the volume of tumor in mouse injected with HepAD38 without tetracycline (HBV expression) increased gradually compared to the control mouse (no HBV expression) (Figure 7a). Mice were sacrificed 4 weeks after the SC injection and the volume and weight of tumor were examined. The weight and the volume of tumor were almost doubled in mice with HBV-expressing HepAD38 xenograft (without tetracycline) (Figure 7b,c). The level of HNF4α was dramatically suppressed in the mice bearing the HepAD38 xenograft maintained in the absence of tetracycline (Figure 7d), confirming the HBV-induced inhibition of HNF4α in mice after 4 weeks of injection. Similar results were obtained with the HepG2-X xenograft (Figure 7e). The tumor volume was measured up to 39 days post SC injection. Between days 24 and 39, the tumor volume continuously expanded and tripled in size, reaching more than 600 mm^2^ and weighed 400 mg at day 39 (Figure 7f,g). Furthermore, the level of HNF4α was suppressed in HepG2-X xenografts in nude mice when measured at 5 weeks post injection, as compared to the mouse having a HepG2-pc xenograft (Figure 7h). These data also suggest that HBV infection downregulates HNF4α and enhances tumorigenesis in vivo.

## 3. Discussion

HNF4α was identified as a nuclear factor which exists abundantly in the liver and controls the transcription of many genes involved in hepatocyte function [9,11]. Different roles of HNF4α have been described, including the tumor suppressive ability that inhibits tumor progress in HCC [10,13,25].

HBx protein plays an important role in HBV replication and liver pathogenesis through regulation of different host proteins that are involved in cell differentiation and proliferation [6,26,27,28,29,30]. It has been reported that HBx modifies the epigenetic regulation of HBV by interaction with cccDNA, which serves as a template for HBV replication [31]. Therefore, it is crucial to unveil the exact mechanism of HBx-related carcinogenesis in order to inhibit HBV-related liver failure. 

The inhibition of hepatocyte proliferation and tumor growth by HNF4α have been well characterized in mice and human tissues [10]. Expectedly, HNF4α suppression has been reported in cancers, including HCC [13,25]. Activation of AKT by the HBx protein downregulates HNF4α transcription in rat hepatocyte and is suggested to contribute to the development of HBV-associated HCC [14,32]. In addition to the AKT signaling pathway, HBx can regulate ERK through the Notch1 pathway in HCC [14,20]. 

There has been no previous report on HBx-mediated downregulation of HNF4α in human hepatoma cells. Moreover, the effect of other cell signaling pathways, including ERK on HBV-mediated HNF4α suppression, have not been explored. Previous studies reported that serum from CHB patients enhanced cell growth and proliferation in HCC cells via the IGF-II/IGF-IR/MEK/ERK signaling pathway [33]. Moreover, HBx, through the activation of the ERK and p38 MAPK signaling pathways, promoted the metastasis of liver cancer [34]. Here, we showed that transcriptional regulation of HNF4α is a target of HBx-mediated ERK activation in both human and mouse hepatocytes.

In this report, we demonstrated that HBV-induced downregulation of HNF4α could enhance the proliferation of hepatoma cells and increase their tumorigenicity both in vitro and in vivo. Of note, HNF4a recovery through a pharmacological or genetical approach greatly contributed to the inhibition of cell proliferation. Moreover, our results clearly reveal the substantial efficacy of HBV expression on liver tumor progression in animal models. Therefore, HBV-mediated downregulation of HNF4a plays a considerable role in HBV-related carcinogenesis. Although others have shown that HBx activates signals that can diminish the overall level of HBV replication in order to balance cell survival [14], in long-term HBV infection, we showed a considerable reduction in HNF4α level attained by HBx to elevate cell proliferation and tumor progress. 

In the present study, by arresting several signaling pathways, we showed that the suppressed level of HNF4α by HBV was only restored in the presence of U0126, an ERK signaling inhibitor. There was no alteration in the level of HNF4α when inhibitors for AKT, JNK, p38 and mTOR were applied. Unlike the previous report, which suggested that the AKT signaling pathway is associated with the downregulation of HNF4α in rat primary hepatocyte [14], our results here reveal that the suppression of HNF4α during long-term HBV infection is associated with the ERK signaling pathway in human hepatoma cell lines and mouse models. Similarly to HBV genome expression, ectopic expression of HBx itself could induce the same diminishing effect on HNF4α in the HepG2 cell. Notably, inhibiting the ERK signaling pathway restored HNF4α protein level in the HepG2-X cell line.

We also evaluated the effect of different HBV genotypes on HNF4α level in vivo. The expression level of HNF4α was suppressed in long-term infection of HBV in the mouse model. Importantly, in mice infected with HBV genotype A, B or C, regardless of genotype variation, the HNF4α level was plunged at six weeks post infection. In this study, even though the HNF1α level was slightly decreased in HepG2.2.15 and HepAD38 cells as a result of HNF4α reduction, there were no significant alteration in the HNF1α level in mice data (Figure 4c). Further study is required to understand the exact correlation between HNF4α and HNF1α in mice infected with HBV in the long term.

Many findings implicate HBV infection as a major risk factor in the development of HCC [33,35]. Here, we demonstrated that prolonged HBV infection in susceptible host cell line (HepG2-NTCP) or in long-term infected mice can suppress the expression of anti-tumor HNF4α, particularly at later time points. This notion is in line with the fact that HCC occurs with a slow pace in HBV-infected patients and virus-induced tumorigenesis is mostly observed at late stages of infection [3,36,37]. Perhaps epigenetic modifications in HNF4α suppressed cells during long-term HBV infection may contribute to liver carcinogenesis. 

Overall, the results of our study with cultured human hepatocytes and in vivo mouse models demonstrate that HBx activation of the ERK1/2 signaling pathway downregulates HNF4α and consequently contributes to the tumorigenesis in HBV infected liver.

## 4. Materials and Methods 

### 4.1. Cell Culture

Human hepatoma cell lines (HepG2, HepG2.2.15, HepAD38, HepG2-pc, HepG2-X and HepG2-NTCP-K7) were grown in Dulbecco’s Modified Eagle’s medium (DMEM) containing 10% heat inactivated Fetal Bovine Serum (Capricorn; Ebsdorfergrund, Germany) and 1% of penicillin-streptomycin (Gibco; BRL, Grand Island, NY, USA) at 37 °C in 5% CO_2_. The HepG2-NTCP-K7 cell line was kindly provided by Professor Ulrike Protzer (Institute of Virology, Munich, Germany).

### 4.2. Plasmid Construction

The constructs for WT HBV 1.2mer (1.2(+)), X-null HBV 1.2mer (1.2(-)), HBV 1.3mer (1.3(+)) and X-null HBV 1.3mer (1.3(-)) were kindly provided by Wang Shick Ryu [38,39] (Yonsei University, Seoul, South Korea). The construct of HBx-HA was described in a previous study [28]. The plasmids for pAAV control vector and pAAV-HBV genotype A, B, and C were kindly provided by Professor Pei-Jer Chen (National Taiwan University, Taipei, Taiwan) [40]. In order to make HNF4α expressing construct, the full sequence of HNF4α was cloned into the pcDNA3.1(+) vector (Invitrogen, CA, USA).

### 4.3. Animal Experiment 

Hydrodynamic liver transfection of pAAV vector and pAAV-HBV were performed in C57B/L6 mice (6 weeks old) with transfection of 12.5 μg of plasmids. The plasmid DNA was injected into the tail vein with high pressure within 3-6 s. The mice were sacrificed after 1 week or 6 weeks of DNA injection. Recombinant AAV-HBV construct (rAAV-HBV) expressing AAV8 serotype was prepared from pAAV-HBV plasmid and was injected into the mice through intravenous injection (IV). All animal experiments were conducted with the approval of Institutional Animal Care and US Committee (IACUC), Konkuk University (KU15049, 27th April 2015). 

### 4.4. Transfection and Reagents 

Transient transfection was performed at 70–80% of cell confluency, using Lipofectamine 2000 reagent (Invitrogen, Carlsbad, CA, USA) according to the manufacturer’s instruction manual. The cells were harvested 2 days after transfection.

Signaling pathway inhibitor molecules, U0126, LY294002, SP600125, SB203580, Rapamycin were purchased from Cell Signaling Technology, (Danvers, MA, USA). The U0126, LY294002, and SP600125 were treated at 10 μM, SB203580 was treated at 20 μM, and rapamycin was treated at 100 mM, respectively.

### 4.5. Virus Particle Production

For HBV particle production, HepAD38 cells were cultured in large scale with media containing tetracycline (500 ng/mL) as described previously [41]. Cells were transferred to a 5-layer T175 flask in tetracycline free media for 2 weeks to induce virus expression. Subsequently, the supernatant was collected every three days, centrifuged at 6000 rpm and filtered using 0.22 μm pore size filters. Virus particles in the collected supernatant were concentrated overnight at 4 °C using 6.5% PEG 8000. Concentrated supernatant was centrifuged for 1 h at 10,000 rpm. Pellet was resuspended in 10% FBS/PBS. Virus titration was quantified by dot blot assay. 

### 4.6. Virus Infection

HepG2-NTCP-K7 cells were seeded in 6-well collagen coated plates. HBV infection was performed using cell culture derived inoculum about 2000 Geq/cell per well in a mixture with culture media containing 4% PEG 8000 for 16 h. Cells were washed extensively with PBS. Infected cells were maintained in the media with 2.5% DMSO and harvested as indicated time points. 

### 4.7. Western Blot 

The harvested cells and obtained tissues were lysed by RIPA buffer [20 mM Tris/HCl, 1% NP-40, 0.5% phosphatase inhibitor cocktail (Sigma, St Luis, MO, USA), 150 mM NaCl, 2 mM KCl, pH 7.4]. The debris was removed by centrifuging at 12000 RPM for 20 min. After the filtering of cell debris, levels of protein in the lysate were quantified by a BCA protein assay kit (Thermo Fisher scientific, Waltham, MA, USA). The samples were mixed with 5X SDS sample buffer and boiled at 95 °C for 4 min. The proteins were separated by SDS-PAGE and transferred to polyvinylidene fluoride (PVDF) membrane (Millipore, Burlington, MA, USA). The transferred membranes were washed with TBS-T. Antibodies were used in 1:2000 ratios. The primary antibodies used were as follows: anti-HNF4α (Santa Cruz, CA, USA), anti-HNF1α (Santa Cruz), anti-HNF3β (Santa Cruz), anti-C/EBPα (Santa Cruz), anti-HBcAg (Dako, Glostrup, Denmark), anti-HBx protein (Abcam, UK), anti-p-ERK (Cell Signaling Technology, MA), anti-ERK (Cell Signaling Technology), anti-p-AKT (Cell Signaling Technology), anti-AKT (Cell Signaling Technology), anti-p-JNK (Cell Signaling Technology), anti-JNK (Cell Signaling Technology), anti-p-p38 (Cell Signaling Technology), anti-p38 (Cell Signaling Technology), anti-β-actin (Sigma-Aldrich Co, St Louis, MO, USA).

### 4.8. ELISA

The levels of HBeAg and HBsAg were examined with the ELISA kit (Wantai, Beijing, China) according to the manufacturer’s instructions. For HBeAg and HBsAg measurement, samples were diluted at a 1:10 or 1:100 ratio, respectively. The absorbance values were measured at 450 nm via a microplate reader.

### 4.9. Real-time Quantitative PCR (qRT-PCR)

HepG2, HepG2.2.15, HepAD38 cells were lysed with Trizol reagent (Sigma) according to the manufacturer’s manual. An amount of 2 μg of extracted total RNA was used for the reverse transcription reaction by MMLV reverse transcriptase (Intron Biotechnology, Kyeonggi, Korea) in a final volume of 20 microliter. qRT-PCR was carried out to amplify cDNA by using SYBR Green PCR master mix (Applied Biosystems) in an ABI PRISM 7500 sequence detection machine. 

Relative quantification was performed by the comparative ΔΔCt method [42]. The results were shown as an n-fold difference to calibrator (RQ=2^−ΔΔ*C*t^). The HNF4α gene was amplified using targeting primers; For 5′-GAGTGGGCCAAGTACATCCCAG-3′, Rev 5′-GCTTTGAGGTAGGCATACT-3′

### 4.10. Cell Proliferation and Colony-Forming Assay

For cell proliferation assay, cells were seeded at 2 × 10^5^ cells per well in a 6-well plate or 12-well plate. The number of cells in each well was determined by cell counting using a hemocytometer. Colony forming assay was performed to compare the colony forming abilities of each cell line. Cells were harvested with trypsin-EDTA and resuspended in a singular form. The 1 × 10^4^ cells were plated in a 6-well plate. After incubation for 4 weeks, colonies were stained with 0.5% crystal violet (Sigma) for 30 min at room temperature. 

### 4.11. Soft Agar Assay

To determine anchorage-independent growth of each cell lines, soft agar assay was performed. Cells (1.0 × 10^4^ cells per well) mixed with 0.35% Difco Noble Agar (BD) in complete medium were plated on the top of 0.5% agar layer in a 6-well plate with complete medium. Growth medium (1.5 mL) was added on the top of the layer and the cells were incubated at 37 °C for 4 weeks. For visualization, foci were stained with 0.0005% crystal violet.

### 4.12. Xenograft

HepAD38 (maintained with or without tetracycline) or HepG2-X cell lines were injected into the six-week old male BALB/c nude mice (Nara Biotec, Seoul, Korea). Cells were counted at 1 × 10^7^ in a volume of 100 μL with 1:1 of Matrigel (Corning, NY, USA) and subcutaneously (SC) inoculated into the back of the mice. Mice xenografted with HepAD38 grown with tetracycline were housed with water containing 2 mg/mL tetracycline. The size of tumor was measured every 3 days using a caliper. The tumor volume was calculated by the following equation: tumor volume [mm^3^] = (length [mm]) × (width [mm])^2^ × 0.52. All the measurements were compared using the Student’s *t*-test. The animal procedures were approved by the Animal Care Committee at Konkuk University (KU15049, 27th April 2015).

## Abbreviations

CHB	Chronic Hepatitis B Virus
dpi	Days post infection
Geq	Genome equivalent
HBeAg	HBV e antigen
HBsAg	HBV surface antigen
HBV	Hepatitis B virus
HBx	Hepatitis B virus X protein
HCC	Hepatocellular carcinoma
HNF1α	Hepatocyte nuclear factor 1 α
HNF3β	Hepatocyte nuclear factor 3 beta
HNF4α	Hepatocyte nuclear factor 4 α
IV	Intravenous
NTCP	Sodium-taurocholate cotransporting polypeptide
pAAV-HBV	Plasmid adeno associated virus-hepatitis B virus
rAAV-HBV	Recombinant adeno associated virus-hepatitis B virus
SC	Subcutaneous
Tet	Tetracycline
wpi	Weeks post infection
